# Editorial: Sex hormones and cardiovascular-renal health: mechanisms, consequences, and therapeutic insights

**DOI:** 10.3389/fendo.2026.1933342

**Published:** 2026-07-21

**Authors:** Prerna Kumar, Evangeline Deer, Noha Shawky Elsayed

**Affiliations:** 1 Department of Physiology, Tulane University Health Sciences Center School of Medicine, New Orleans, LA, United States; 2 Department of Pharmacology and Toxicology, Mississippi Center of Excellence in Perinatal Research, Women’s Health Research Center, University of Mississippi Medical Center, Jackson, MS, United States

**Keywords:** cardiovascular, renal, sex difference, sex hormones, women’s health

Sex steroids are known to regulate endothelial function, vascular tone, inflammatory signaling, oxidative stress, and extracellular matrix remodeling, all of which contribute to cardiovascular (CV) risk (1). They also modulate blood pressure (BP), fluid balance, lipid metabolism, and inflammation, contributing to sex-based differences in health and disease. Despite growing recognition of these influences, key molecular mechanisms and clinical implications remain poorly understood. This Research Topic was designed to help better understand the molecular, physiological, and clinical effects of sex hormone signaling on CV and renal health in both sexes across different periods of life.

In women, critical periods of life that are coupled with hormonal changes can shape their future risks of CV disease (CVD) (2). Women are generally accepted to be at lower risk of CVD compared to men during reproductive years of their lives. However, unique sex-specific reproductive and pregnancy history in women that occur in those years can shape their future risks (3). Moreover, during the menopausal transition, women demonstrate characteristic shifts in sex hormone profiles accompanied by adverse alterations in body composition, lipid levels, and vascular function, collectively contributing to an elevated risk of postmenopausal CVD (4). This highlights the critical need to focus more studies on women undergoing menopausal transition and its correlation to the heightened CVD risk. In this context, the study by Wang et al. investigated differences in multiple clinical parameters between perimenopausal women (n = 176) with CVD and postmenopausal women (n = 120) with CVD across different age groups (40-44, 45-49, 50-54). The authors reported that postmenopausal women had significantly higher body mass index (BMI), waist-to-hip ratio, total body fat percentage and distribution, systolic and diastolic BP with no change in lipid profile and oral glucose tolerance compared with perimenopausal women [DOI 10.3389/fendo.2025.1681941]. Peri- and post-menopause are periods where women are at an increased risk of developing metabolic disorders, such as insulin resistance (IR), abdominal obesity and elevated blood sugar, which also increases the incidence of CVD (5). The estimated glucose disposal rate (eGDR) is recognized as a reliable marker of IR that is independently associated with an increased risk of CVD, coronary heart disease (CHD), and stroke. The meta-analysis performed by Zhang et al. provides robust evidence regarding eGDR’s prognostic utility and its continuous relationship with CVD risk [DOI 10.3389/fendo.2025.1740472]. In this meta-analysis, electronic databases such as PubMed, Web of Science and Embase were searched for cohort studies reporting eGDR and CVD risk. The included studies enrolled adult subjects (56 to 63 years; both sexes) without baseline CVD, measured eGDR at baseline and reported hazard ratio, with follow-up durations ranging from 5.6 to 14.1 years. The study reported that higher baseline eGDR levels are associated with a reduced risk of CVD, stroke, and CHD in individuals without preexisting CVD. Subgroup analyses indicated that variables such as sex, sample size, follow-up duration, and prediabetes/diabetes status did not significantly influence the observed associations. Furthermore, dose-response analysis demonstrated a linear inverse relationship between eGDR and CVD risk. Whether changes in eGDR can be utilized as a prognostic marker for CVD in women during menopausal transition is an area that needs further investigation.

The study by Wang et al. was directed towards investigating potential biomarkers that could be used as prognostic tools for left atrial reverse remodeling (LARR) in patients (~65 years; both sexes) with atrial fibrillation (AF) after catheter ablation (CA) [DOI 10.3389/fendo.2025.1717422]. CA for AF is an important rhythm-control strategy in symptomatic patients with paroxysmal or persistent AF who are refractory or intolerant to antiarrhythmic drugs. Corin is a type II transmembrane serine protease highly expressed in cardiomyocytes and converts pro-atrial natriuretic peptide (ANP)/pro-brain natriuretic peptide (BNP) into mature ANP/BNP, and is associated with CVD (6, 7). In the total cohort, the plasma corin concentrations drawn pre- and post-procedure in the LARR group were significantly higher than those in the non-LARR group. However, sex-specific subgroup analysis, male patients with LARR had significantly higher pre-CA plasma corin concentrations than in those without LARR, whereas no differences were observed in post-CA or among female patients, highlighting the importance of the development of sex-specific CVD markers.

Importantly, while plenty of studies examined the role of estrogen and testosterone in CV and renal physiology, the contribution of progesterone signaling to cardiorenal physiology/pathophysiology in females and males remains unclear. Nguyen and Kriegel summarized evidence on the role of progesterone, beyond reproduction, highlighting its importance in cardiorenal health and disease [DOI 10.3389/fendo.2026.1837458]. On the vasculature, progesterone lowers vascular resistance through two mechanisms: increased endothelial nitric oxide production and direct effects on smooth muscle, however its effects on BP remain unclear (8, 9). Additionally, progesterone lacks protective effects in atherosclerotic models when supplied during the development of associated pathology. The authors also summarized the renal effects of progesterone replacement in ovariectomized animal models of kidney disease including its effects on water reabsorption, ion transport, autonomic function, and renal vasculature in order to isolate the progesterone-specific effects on the kidney. Progesterone receptors are expressed in many male tissues. Experimental studies indicate that progesterone has limited effects on renal tubular transporters; sodium glucose transporter1 (SGLT1) and organic anion transporter (OAT)-2, but may upregulate OAT1 expression and confer protection against ischemia-reperfusion-induced kidney injury through anti-inflammatory and antioxidant mechanisms.

Apart from the changes in endogenous hormone levels, exogenous hormonal therapy including gender-affirming hormonal therapy (GAHT). Unfortunately, studies into the long-term consequences of GAHT on CVDs in transgender individuals are lacking (10). Ciancia et al. performed comprehensive echocardiographic assessments in 47 trans men (TM) and 6 trans women (TW) who had undergone GAHT for 5–10 years [DOI 10.3389/fendo.2026.1793245]. Systolic cardiac function remained within normal limits in all participants, while cardiac dimensions and left ventricular mass were within normal reference ranges. Notably, cardiac structural characteristics corresponded to the affirmed gender rather than sex recorded at birth, thereby highlighting the importance of selecting appropriate reference values when assessing CV parameters in transgender individuals. The authors suggest that while cardiac structure and function remain unchanged, prolonged exposure to testosterone or estrogen may influence vascular properties, as evidenced by increased aortic stiffness and reduced vascular elasticity in both TM and TW. Arterial stiffness is recognized as an early marker of vascular aging and an independent predictor of future CV events (11). Therefore, these results demonstrate the possibility of early vascular remodeling associated with long-term hormone exposure, although the clinical significance of these changes remains uncertain. Importantly, exploratory analyses of untreated transgender adolescents did not demonstrate similar abnormalities, suggesting that prolonged GAHT exposure may contribute to these vascular alterations.

In conclusion, the articles included in our Research Topic highlight the role of endogenous and exogenous sex steroids in CV and renal health in men and women and address the need for better sex-specific CVD prognostic tools.
